# Preliminary insights into the potential role of Acanthamoeba–Pseudomonas interactions in the development of antibiotic resistance

**DOI:** 10.1099/acmi.0.000999.v3

**Published:** 2025-06-30

**Keywords:** *Acanthamoeba*, antimicrobial resistance, ciprofloxacin, interkingdom, microbial interactions, multidrug resistance, *Pseudomonas*

## Abstract

Interactions between environmental protists and bacteria play a crucial role in shaping bacterial survival strategies and pathogenic potential. Certain bacteria have evolved mechanisms to resist predation by protists such as *Acanthamoeba*, allowing them to persist intracellularly and, in some cases, enhance their virulence. We hypothesize that *Acanthamoeba* species may also play a role in promoting antimicrobial resistance (AMR) in amoeba-resistant bacteria. This study investigated whether *Acanthamoeba castellanii* enhanced AMR development in *Pseudomonas putida* under lethal ciprofloxacin concentrations. *P. putida* was co-incubated with *A. castellanii* and maintained in ciprofloxacin concentrations starting at 2 µg ml^−1^, four times the planktonic MIC, which was incrementally increased as resistance emerged. The survival of the co-incubated *P. putida* and the development of resistance were monitored, and antimicrobial susceptibility tests were conducted using multiple antibiotics. *P. putida* co-incubated with *A. castellanii* in the presence of ciprofloxacin became increasingly resistant in a dose-dependent manner, with the MIC increasing from 0.5 to 20 µg ml^−1^ after 17 days. Contrastingly, the naïve strain did not survive sustained exposure at 2 µg ml^−1^. Co-incubated bacteria maintained under ciprofloxacin pressure developed resistance to ciprofloxacin, chloramphenicol, azithromycin and enrofloxacin while retaining susceptibility to streptomycin and tetracycline. Co-incubation in the absence of ciprofloxacin did not promote resistance in *P. putida*, suggesting that the combination of extracellular drug pressure and intracellular survival is important in driving resistance. These findings indicate that intracellular survival within *Acanthamoeba* can significantly accelerate AMR development in * P. putida* under fluoroquinolone pressure. Further research into the molecular mechanisms involved is warranted to inform strategies for mitigating AMR emergence in clinical and environmental contexts.

**Impact Statement**

This study provides new insights into the role of *Acanthamoeba* in shielding bacteria from lethal antibiotic conditions. Experimental findings show that *Acanthamoeba castellanii* protects *Pseudomonas putida* by enabling survival at ciprofloxacin concentrations exceeding its MIC of 0.5 µg ml^−1^. *P. putida* co-incubated with *A. castellanii* survived ciprofloxacin concentrations up to 20 µg ml^−1^, which is forty times the MIC for the naïve strain (0.5 µg ml^−1^). In contrast, the naïve strain was completely inhibited at 2 µg ml^−1^, demonstrating the critical protective role of *Acanthamoeba* in enhancing bacterial resistance.

The study further reveals that co-incubation drives the development of antimicrobial resistance (AMR). The co-incubated *Pseudomonas* strain exhibited progressive resistance, with MIC increasing incrementally from 4 to 20 µg ml^−1^ as ciprofloxacin pressure increased. Additionally, these strains developed resistance to multiple antibiotics, including chloramphenicol, azithromycin and enrofloxacin, while retaining susceptibility to streptomycin and tetracycline. These findings emphasize *Acanthamoeba*’s dual role as a protective reservoir and facilitator of AMR, highlighting its importance in public health and antibiotic resistance research. The results point out the urgent need to address microbial interactions that exacerbate the AMR crisis and inform global mitigation strategies.

## Data Summary

The authors confirm that all supporting data, code and protocols have been provided within the article or through supplementary data files.

## Introduction

*Acanthamoeba* spp. are free-living, ubiquitous amoebae isolated from a wide range of environments [1,2] which survive by predating on other micro-organisms, such as bacteria and fungi, via phagocytosis [3]. This predation has resulted in the emergence of various amoeba-resistant micro-organisms, including *Pseudomonas* spp., which can resist phagocytosis and survive intracellularly [4]. *Pseudomonas* spp. can evade destruction within *Acanthamoeba*’s phagosomes and are capable of replication inside the trophozoites [5]. Moreover, they possess the ability to recover from a viable but non-culturable state upon contact with the amoeba, reverting to an active form under favourable conditions [6]. Amoeba-resistant *Pseudomonas* are protected from environmental stresses, allowing them to persist and survive in hostile conditions [3]. This association is particularly concerning in clinical settings, where it facilitates the bacteria’s evasion of detection and disinfection, thus complicating infection control in environments such as hospital water systems [7].

Studies have shown that *Pseudomonas aeruginosa* can adapt to internalization by *Acanthamoeba* through mutations that result in reduced virulence while increasing fitness and survival within amoebae and other host cells [8,9]. Mutations in the *lasR* gene, which regulates virulence factors, are common in both amoeba-resistant isolates and chronic infections, but these mutations do not always lead to decreased virulence [10,11]. Additionally, the type III secretion system, including components like the PscJ protein and cytotoxins such as ExoU, plays a crucial role in maintaining *P. aeruginosa* virulence against amoebae, enabling the bacterium to survive and replicate within these protozoa [10]. Despite attenuated virulence, *P. aeruginosa* maintains enhanced resistance to phagocytosis and persists within host cells [8,12]. The ubiquity of *Acanthamoeba* and its association with amoeba-resistant bacteria such as *Pseudomonas* spp. highlights the importance of understanding these interactions to control the spread of pathogenic bacteria in hospital settings [13]. Within the *Pseudomonas* genus, species such as *P. aeruginosa* are well-established opportunistic pathogens, particularly in immunocompromised individuals [14]. Other species, including *Pseudomonas putida*, *Pseudomonas fluorescens,* and *Pseudomonas mendocina*, have also been implicated in human infections, though these occurrences are less common and typically opportunistic in nature [15,17].

Ciprofloxacin is an antibiotic used to treat infections caused by *Pseudomonas* and is effective against multiple antibiotic-resistant strains of this pathogen [18,19]. It primarily targets bacterial DNA gyrase, a type II topoisomerase essential for DNA replication and transcription; by inhibiting DNA gyrase, it disrupts the supercoiling of bacterial DNA, leading to the cessation of these processes and ultimately causing bacterial cell death [20,21]. The bactericidal action of ciprofloxacin is rapid and can lead to a post-antibiotic effect, in which bacterial growth is suppressed even after the drug is removed, although regrowth can occur [22,23]. However, *Pseudomonas*’s ability to acquire mutations that confer resistance to ciprofloxacin and other antimicrobial agents poses a significant challenge [24]. According to the current European Committee on Antimicrobial Susceptibility Testing (EUCAST) and Clinical and Laboratory Standards Institute (CLSI) guidelines, ciprofloxacin remains a fluoroquinolone deemed effective against *Pseudomonas*, but the increasing prevalence of antibiotic-resistant strains raises significant concerns [25,26]. The rise of multidrug-resistant bacteria, driven by factors such as antibiotic misuse in human medicine and agriculture and the presence of antibiotic-resistance genes in the environment, has become a global public health issue [27,28]. *Acanthamoeba* species have been shown to protect co-incubated bacteria in the presence of antimicrobial agents such as hydrogen peroxide [7], and we hypothesize that *Acanthamoeba* will protect bacteria against other antimicrobial agents, such as antibiotics. This study aimed to investigate the role of *Acanthamoeba* in protecting *Pseudomonas* when exposed to lethal concentrations of ciprofloxacin and to establish whether amoeba-associated *P. putida* develop antimicrobial resistance (AMR) traits after prolonged exposure compared with a naïve laboratory strain. These findings provide insights into the interkingdom relationship that can drive AMR and contribute to developing strategies for combatting antibiotic-resistant bacteria in environmental and clinical settings.

## Methods

### *Pseudomonas* and *Acanthamoeba* strains used in this study

The bacterial strain *P. putida* KT2440 plasmid-free donor strain [29], kindly donated by Uli Klümper, at a density of 10⁵ c.f.u. ml^−1^, and *Acanthamoeba castellanii* ATCC 50370, at a density of 5×10⁴ cells ml^−1^, were utilized in this study. *P. putida* KT2440 was selected due to its relevance as an environmental organism with emerging opportunistic pathogenicity. Crucially, in contrast to *P. aeruginosa*, *P. putida* exhibits lower intrinsic resistance to antibiotics, making it an ideal model for studying *de novo* resistance acquisition under selective pressure and allowing clearer attribution of resistance phenotypes to experimental conditions.

### MIC and reference values for ciprofloxacin

A stock solution of ciprofloxacin (≥98% purity, HPLC grade, Sigma-Aldrich) was prepared by dissolving 100 mg of the antibiotic in 1 ml of acetic acid (CH_3_COOH) (99.8% purity, for analysis, Acros Organics) before further diluting in ddH_2_O to a final concentration of 1 mg ml^−1^. A range of concentrations was selected for testing based on standard guidelines and previous literature, where the MIC for *Pseudomonas* was established at 0.5 µg ml^−1^ [25,26]. Ciprofloxacin was tested at concentrations of 0.02 µg ml^−1^, 0.05 µg ml^−1^, 0.1 µg ml^−1^, 0.5 µg ml^−1^, 1 µg ml^−1^, and 5 µg ml^−1^ to cover the spectrum of potential MIC for the selected *Pseudomonas* species.

A 24-well plate was used for this assay. Luria–Bertani (LB) broth (Sigma®), prepared according to the manufacturer’s instructions, was used as the growth medium and 1 ml of broth was added to each well. Each well received an appropriate concentration of ciprofloxacin and a bacterial inoculum of 10^5^ c.f.u. ml^−1^. The positive controls consisted of bacteria (10^5^ c.f.u. ml^−1^) in LB broth, whereas the negative controls consisted of LB broth without bacteria. A solvent control plate was used to ensure no effect of acetic acid on bacterial growth at the dilution factors noted above. The inoculated plates were incubated in a shaking incubator (0.34 g) (Grant Bio ES-80) at 37 °C overnight. After 24 h, the OD of the cultures was measured at a wavelength of 595 nm using a Tecan Infinite 200 PRO spectrophotometer (Tecan Austria GmbH). The MIC reference values were established based on the experimental results and in accordance with the EUCAST guidelines, which set the resistance breakpoint for ciprofloxacin at 0.5 µg ml^−1^ [25].

### Evaluating *A. castellanii*-induced antimicrobial resistance in *P. putida*

*A. castellanii* ATCC 50370 trophozoites were maintained in peptone glucose (PG) medium [7]. PG medium containing 5×10^4^ cells ml^−1^ was added to a 96-well plate to a final volume of 200 µl, and amoebae were left to adhere to the bottom for 40 min. *P. putida* KT2440 (10^5^ c.f.u. ml^−1^) was added to the experimental and positive control wells, resulting in a bacteria-to-amoeba ratio of 2:1. The amoebae were given 1 h to ingest the bacteria before 2 µg ml^−1^ of ciprofloxacin (Sigma-Aldrich) was added to the experimental wells. Inoculated plates were placed in a shaking incubator (0.34 g) (Grant Bio ES-80) overnight at 25 °C. After 24 h, absorbance was measured at 595 nm using a Tecan Infinite 200 PRO spectrophotometer (Tecan Austria GmbH). Once the bacterial OD of all experimental wells exceeded 0.1 (595 nm), 10 µl of bacteria were transferred into a centrifuge tube with LB broth and the corresponding experimental antibiotic concentration and left to grow in a shaking incubator at 37 °C (0.34 g) overnight. To control for background absorbance from *Acanthamoeba*, amoeba-only wells were included in each assay as blanks, and their OD_595_ values were subtracted from co-incubation readings. OD (595 nm) was measured and adjusted to a 0.1 value, 10^5^ c.f.u. ml^−1^ were then added to a new well with *A. castellanii* and the process was repeated with an increased drug concentration. Overall, the process was repeated with incremental concentrations of ciprofloxacin (3, 4, and 5 µg ml^−1^), and the OD_595_ was monitored daily at 24 h intervals.

A further 96-well plate was prepared using only bacteria at the same antibiotic concentrations. The positive controls consisted of 200 µl of a bacterial solution containing amoebae, bacteria and media only, while an additional control using *P. putida* passaged three times alongside *A. castellanii* without ciprofloxacin was screened to ensure resistance development was due to antibiotic exposure and not intracellular survival. The negative controls consisted of 200 µl medium with and without antibiotics. The experiment was conducted with four biological replicates, and all experimental conditions were tested in triplicate. The purity of the assessed cultures was monitored daily via Gram staining, comparing the co-incubated bacterial cells with the naïve strain of *P. putida* KT2440 to monitor for contamination. The term ‘naïve strain’ refers to the original *P. putida* KT2440 strain that was cultured in LB broth without exposure to either *A. castellanii* or ciprofloxacin. This strain served as a reference for comparative analysis of MIC and AST profiles.

### Evaluation and comparative analysis of AST and MIC in co-incubated and naïve *P. putida*

Upon reaching sufficient bacterial growth, MIC and antibiotic susceptibility testing (AST) was performed in triplicate for each biological replicate. Initial ciprofloxacin concentrations for incubation were set at 2 µg ml^−1^, 3 µg ml^−1^, 4 µg ml^−1^, and 5 µg ml^−1^, with OD_595_ monitored daily at 24 h intervals. During these stages of co-incubation, MICs were tested at incremental concentrations of ciprofloxacin to assess bacterial resistance. The MIC values set for each stage were doubly diluted as follows: 16–0.1 µg ml^−1^ in the first series of tests, 24–0.5 µg ml^−1^ in the second series, 32–1 µg ml^−1^ in the third series and 40–2 µg ml^−1^ in the fourth series.

The AST was conducted on agar plates according to the Kirby–Bauer Disk Diffusion Susceptibility Test Protocol as described in Hudziki [30]. The test guidelines adhered to the standards set by EUCAST and CLSI [25,26]. The antibiotics tested included ciprofloxacin (CIP, 10 µg), streptomycin (S, 25 µg), azithromycin (AZM, 15 µg), enrofloxacin (ENR, 5 µg), chloramphenicol (C, 30 µg) and tetracycline (TE, 30 µg). Antibiotic concentrations were selected based on the standard guidelines outlined by EUCAST and CLSI. The concentration of ciprofloxacin used was 10 µg, which exceeds the reference concentration outlined in the EUCAST and CLSI guidelines (5 µg). This variation may provide valuable insights into the efficacy of antibiotics at higher concentrations, offering a comprehensive assessment of their antimicrobial activity. The naïve *P. putida* KT2440 strain was used to compare the breakpoint diameter inhibition zones of the co-incubated bacteria.

### Statistical analysis

Statistical analysis was conducted using RStudio version 2024.04.1+748 (Chocolate Cosmos Release) in a Windows environment for MIC work. The analysis utilized several R packages, including readxl for data import, dplyr and tidyr for data manipulation. To compare MIC values between co-incubated and naïve *P. putida* KT2440 cultures, the Mann–Whitney *U* test (two-tailed, non-parametric) was applied. To evaluate and compare AST, Welch’s two-sample t-test was applied. A significance threshold of *P*<0.05 was considered statistically significant, with additional significance levels of *P*<0.01 and *P*<0.001 applied where appropriate.

## Results

### Selection of ciprofloxacin-resistant *P. putida* is possible at concentrations above the MIC in the presence of *Acanthamoeba*

**Fig. 1.**
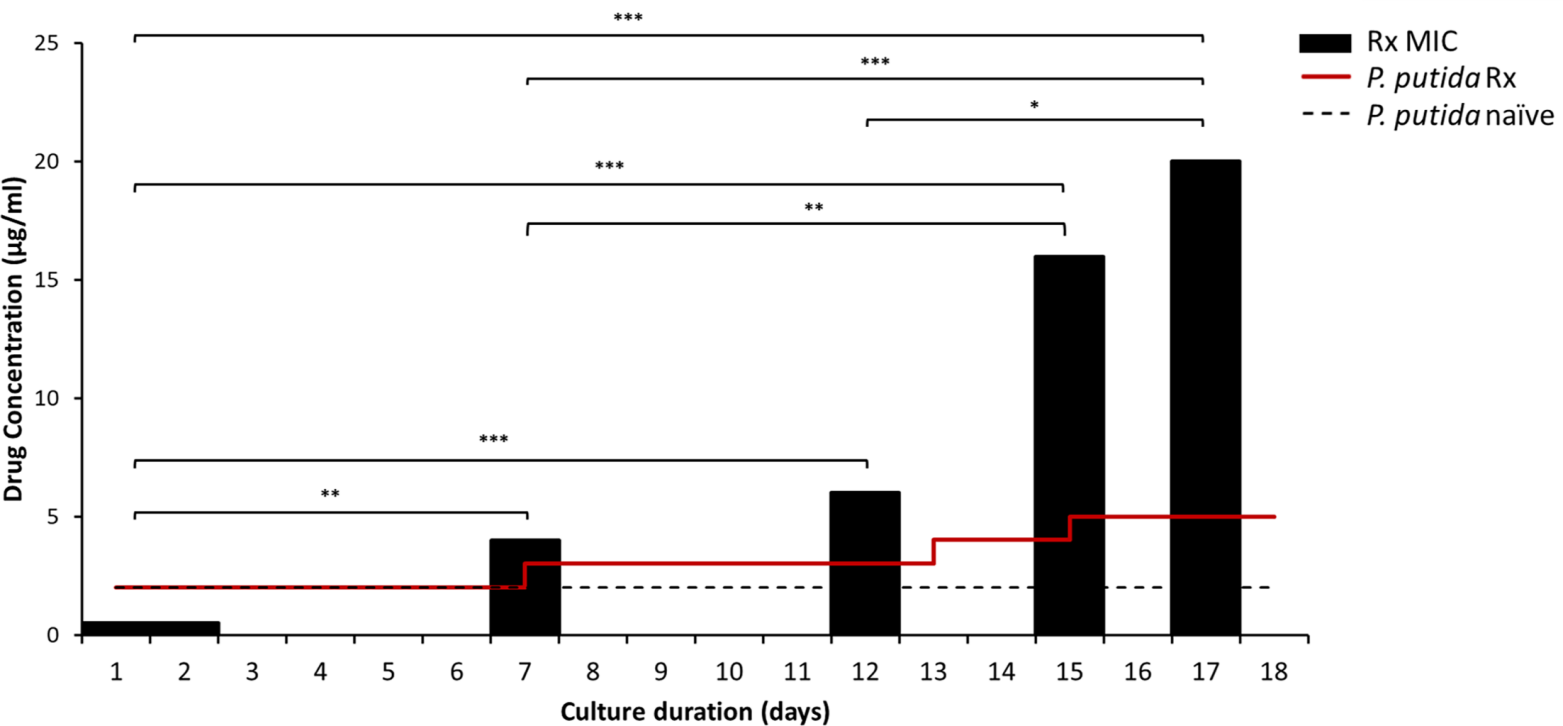
Resistance to ciprofloxacin by *P. putida* KT2440 occurs at higher concentrations of ciprofloxacin and presents higher MIC values when co-incubated with *A. castellanii* ATCC50370. *P. putida* KT2440 (10^5^ c.f.u. ml^−1^) incubated alone (dashed line) or in the presence of 5×10^4^ cells ml^−1^ *A. castellanii* trophozoites (solid red line) were maintained under constant ciprofloxacin pressure, increased incrementally from 2 µg ml^−1^ upon exceeding the established threshold (OD_595_>0.1). Survival at concentrations ten times higher than the established MIC (0.5 µg ml^−1^) is possible after 15 days of incubation with *Acanthamoeba*, while growth was not detected at any point during the experiment for bacteria incubated alone. MICs of resistant bacteria (Rx MIC; black bars) were taken after each increment, with the final increment exhibiting an MIC 40 times higher than that of *P. putida* not passaged within *Acanthamoeba* (naïve strain). The experiment was conducted four times in triplicate. No variation in MIC values was observed between biological replicates at any stage of the experiment; therefore, error bars are not shown. Statistical analysis was performed using the Mann–Whitney *U* test, with significance levels denoted as ‘*’, ‘**’ and ‘***’ for *P*<0.05, *P*<0.01 and *P*<0.001, respectively.

*P. putida* KT2440 was co-incubated with *A. castellanii* ATCC50370 in the presence of ciprofloxacin, starting at a concentration four times higher (2 µg ml^−1^) than the established MIC (0.5 µg ml^−1^) over a period of 18 days (Fig. 1). The results demonstrate that bacteria incubated in the presence of *Acanthamoeba* can survive at antibiotic concentrations higher than those used to inhibit planktonic cells, exceeding the OD_595_ threshold (0.1) within 7 days. Moreover, after 7 days, the MIC significantly increased eightfold (4 µg ml^−1^ vs. 0.5 µg ml^−1^) relative to naïve cultures (*P*<0.01). Although the co-incubated bacterial cells required 7 days to exceed the established threshold in the presence of the initial antibiotic concentration, they demonstrated an increased rate of adaptability to subsequent concentrations of 3, 4, and 5 µg ml^−1^ ciprofloxacin, resulting in a corresponding reduction in the time taken to reach the OD_595_ threshold of 5, 3, and 2 days. Additionally, the minimum inhibitory concentration progressively increased as the bacterial cells adapted to these concentrations, with bacteria maintained at 5 µg ml^−1^ of ciprofloxacin showing a 40-fold increase in MIC relative to naïve cells (20 µg ml^−1^ vs. 0.5 µg ml^−1^, *P*<0.001). In contrast, the naïve *P. putida* KT2440 strain without *Acanthamoeba* was completely inhibited at the initial concentration of 2 µg ml^−1^ for the duration of the experiment.

### Amoeba-resistant *P. putida* develop a distinctive antimicrobial resistance profile relative to planktonic cells

**Fig. 2.**
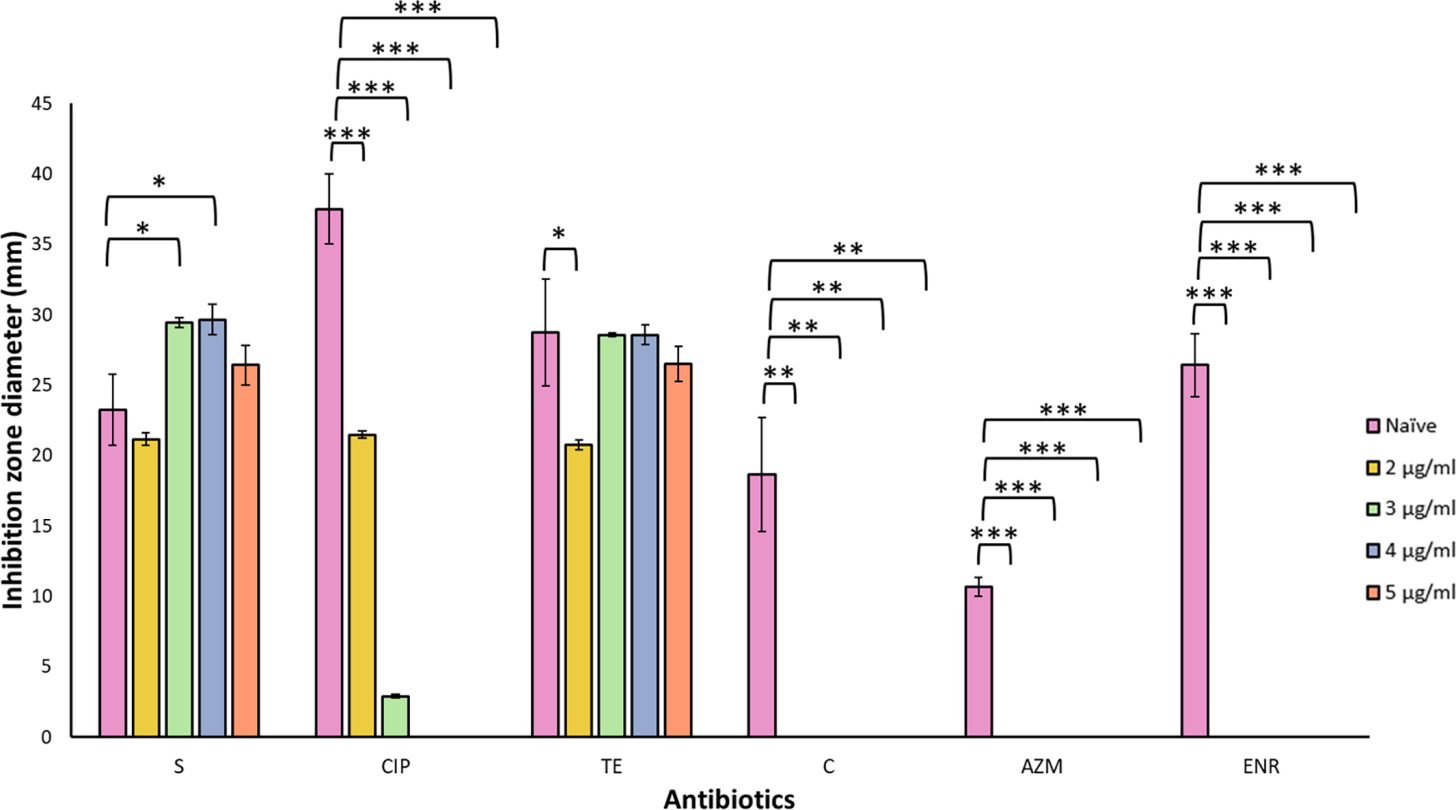
Resistance profiles of *P. putida* KT2440 not passaged within *Acanthamoeba* (naïve strain) and *Acanthamoeba*-associated strains change as ciprofloxacin pressure increases. *Acanthamoeba*-associated *P. putida* KT2440 exhibit a distinct antimicrobial resistance profile compared to the naïve counterpart, with a progressive reduction in inhibition zone diameter corresponding to increasing ciprofloxacin concentrations. Statistical analysis was performed using Welch’s t-test, with significance levels denoted as ‘*’, ‘**’ and ‘***’ for *P*<0.05, *P*<0.01 and *P*<0.001, respectively. Error bars represent sd. Significant differences between the two bacterial groups were observed at most ciprofloxacin concentrations. Data are presented as mean±sd from four independent experiments, each performed in triplicate.

To examine whether gradual exposure to increasing concentrations of ciprofloxacin and the co-incubation passage affected the AMR profile of *P. putida* KT2440, antimicrobial susceptibility tests were conducted in triplicate for each of the four replicates. The antibiotics chosen were based on their efficacy against naïve *P. putida* KT2440 as determined using visible inhibition zone diameters. However, it is worth mentioning that the inhibition zone diameter corresponding to azithromycin was below the susceptibility threshold, as the values ranged between 12 and 10 mm. In contrast, susceptibility was established for values of ≥13 mm [26]. AST results showed that *P. putida* co-incubated with *A. castellanii* and ciprofloxacin developed resistance to ciprofloxacin, chloramphenicol, azithromycin and enrofloxacin relative to the naïve strain (Fig. 2; *P*<0.01 for chloramphenicol, *P*<0.001 for ciprofloxacin, azithromycin, and enrofloxacin). Furthermore, Fig. 2 shows that the inhibition zone diameter for ciprofloxacin progressively decreased in correspondence with the maintained drug pressure of the co-incubated *P. putida*. The antibiotic-free co-cultured bacteria showed no significant difference in their resistance to any antibiotic tested except tetracycline, to which they showed a slight decrease in inhibition zone diameter relative to the naïve cell line (Fig. S1, available in the online Supplementary material; *P*<0.05), albeit remaining susceptible. A similar decrease was noted in the co-cultured *P. putida* exposed to 2 µg ml^−1^ ciprofloxacin (Fig. 2; *P*<0.05). The inhibition zone diameter for streptomycin was wider for the co-incubated bacteria maintained in 3 µg ml^−1^ and 4 µg ml^−1^ ciprofloxacin than that of the naïve lab strain. However, no significant differences were observed in the 2 µg ml^−1^ and 5 µg ml^−1^ co-incubated bacteria (2 µg ml^−1^=*P*>0.05; 3 µg ml^−1^=*P*<0.05; 4 µg ml^−1^=*P*<0.05; 5 µg ml^−1^=*P*>0.05) and all conditions remained susceptible.

## Discussion

In this study, we examined the induction of antibiotic resistance in *Pseudomonas* co-incubated with *Acanthamoeba* and exposed to ciprofloxacin at concentrations exceeding the established MIC of 0.5 µg ml^−1^. We also assessed changes in the AMR profile of the internalized bacteria. To do so, *P. putida* KT2440 was co-incubated with *A. castellanii* and ciprofloxacin was added at a starting concentration of 2 µg ml^−1^, four times the MIC value. The results showed that, while the free-living bacteria did not show any signs of growth for the entire duration of the experiment, the co-incubated bacteria developed resistance within 7 days. It was also observed that the time taken to exceed the OD_595_ threshold (0.1) under each antibiotic concentration decreased as the selective concentration increased. A similar observation was reported by Spagnolo *et al.*, in which streptomycin resistance was induced in several strains of *E. coli*, starting with sublethal antibiotic concentrations [31]. Resistant mutants were first observed within 48 h, and by 96 h, they had become the dominant population; the entire study spanned 7 days, during which resistance progressively increased [31]. Another study that assessed the induction of antibiotic resistance in *E. coli* using chloramphenicol, doxycycline and trimethoprim observed that collectively, it took 20 days for the bacteria to adapt [32]. Bacteria can present a long lag phase as a strategy to limit the damage caused by exposure to antibiotics and gradually develop resistance [33]. While our findings suggest the potential involvement of a lag-time-related strategy based on the division times of *P. putida* KT2440 co-incubated with *Acanthamoeba* compared to its naïve strain, further validation is required.

Evaluating bacterial adaptation to antibiotic exposure and observing how they evolve by maintaining selective pressure are fundamental for understanding how fast bacterial mutants can develop in environmental and clinical settings [34]. Although using sub-MIC antibiotic concentrations has the power to select for resistant mutants [35], the significance of the present study was determined by the initial antibiotic concentration set above the MIC. This theory posits that the selective pressure for resistance is strongest when the antibiotic concentration is above the MIC because this eliminates the growth of susceptible cells, thereby providing a growth advantage for resistant mutants [34,35]. Nevertheless, because single-step mutants usually possess only slightly elevated MICs, they are unlikely to multiply when the antibiotic concentration surpasses a threshold known as the mutant prevention concentration (MPC) [34,35]. The fact that planktonic *Pseudomonas putida* KT2440 failed to survive at the initial 2 µg ml^−1^ ciprofloxacin concentration supports the hypothesis that this value could be determined as the MPC. However, the survival of its co-incubated counterpart underlines the role of *Acanthamoeba* in the emergence of resistant populations. Furthermore, because of the progressing resistance to increasing concentrations of ciprofloxacin, *Pseudomonas* in co-culture with *Acanthamoeba* presented a final MIC value that was 40-fold higher than that of its naïve counterpart. This result contrasts with the observations of a previous study, in which *E. coli* was cyclically exposed to progressively increasing doses of ampicillin [33]. In this case, the MIC of the ancestral strain and mutant clones showed no difference [33]. However, a key difference between this study and ours is that the bacteria were tested as free-living cells and not in co-incubation with *Acanthamoeba*. The intracellular passage and presence of high antibiotic concentrations may have triggered different survival mechanisms, leading to higher antibiotic resistance levels. Previous studies have investigated changes at the DNA level in *P. aeruginosa* co-incubated with *Acanthamoeba* for 42 days [9]. The adapted intra-amoebic *Pseudomonas* population presented non-synonymous nucleotide polymorphisms corresponding to genes involved in coenzyme metabolism, lipid metabolism, cell motility and secondary metabolite production [9]. The accumulation of SNPs was also observed in *Mycobacterium avium* co-incubated with *Acanthamoeba lenticulata* 24 h post-infection [36].

To determine the presence of mutations that led to the high MIC values observed in the bacteria adapted to the ciprofloxacin concentration of 5 µg ml^−1^ in co-incubation with *Acanthamoeba*, comparative genomic and transcriptomic analysis will be needed to provide more insights into the changes at the genomic level induced by the co-incubation passage in the presence of high antibiotic concentrations. Furthermore, such an analysis would help to determine whether the co-incubated *P. putida* KT2440 became resistant, for example, through mechanisms such as altered drug targets, reduced drug uptake, or increased drug efflux [37,38] or simply became more tolerant to antibiotics [37,38], existing in a transient state of reduced susceptibility [39,40]. Further work is required to determine the exact nature of resistance within our *P. putida* strains and the longevity of this resistance. It remains unclear how long *P. putida* can maintain this resistance level, particularly in the absence of *Acanthamoeba*. However, the potential for resistance to emerge above planktonic MICs is highly concerning and warrants further investigation [41,42].

The resistance profiles of *P. putida* naïve and co-incubated strains showed significant changes following intracellular passage with ciprofloxacin, but not without it. Reduced inhibition zone diameters for ciprofloxacin, chloramphenicol, azithromycin, and enrofloxacin suggest resistance development, while cultures remained susceptible to streptomycin and tetracycline. Ciprofloxacin resistance was likely involved with efflux pump overexpression or mutations in quinolone resistance-determining regions (QRDR), reducing binding affinity to DNA gyrase or topoisomerase IV [43,48]. Intracellular environments may facilitate efflux pump overexpression due to stress conditions such as limited nutrient availability or oxidative stress, which trigger adaptive bacterial responses [49,50]. These responses may increase efflux pump production or alter membrane permeability, enhancing resistance to multiple antibiotics [51,53]. Furthermore, intracellular bacteria often experience shifts in membrane potential due to host-derived stressors. These shifts may optimize the activity of proton motive force (PMF)-dependent efflux pumps, amplifying antibiotic extrusion [54]. Resistance to chloramphenicol and azithromycin likely involves efflux mechanisms, while immediate enrofloxacin resistance, compared to progressive ciprofloxacin resistance, could be attributed to specific QRDR mutations or varying efflux pump affinities [53,55]. The retained susceptibility to streptomycin suggests collateral sensitivity linked to PMF-dependent mechanisms. We observed that cultures remained susceptible to tetracycline, suggesting that the mechanisms conferring ciprofloxacin resistance did not lead to cross-resistance. Further investigations are necessary to elucidate the precise molecular mechanisms underlying these resistance profiles. Additionally, while no differences in resistance were observed between the naïve cultures and those passaged alongside *Acanthamoeba* in the absence of ciprofloxacin, it is important to consider the absence of extracellular selective pressure in these conditions. This likely reduces the intracellular duration within the amoebae compared to cultures maintained under continuous ciprofloxacin exposure and may not accurately model the influence of prolonged intracellular survival on the development of antimicrobial resistance. Nonetheless, the lack of observable divergence after three passages suggests that extracellular ciprofloxacin pressure was a major driving factor in the emergence of resistance within our cultures.

The development of antimicrobial resistance is a significant public health concern as it undermines the efficacy of antibiotics and other antimicrobial agents used to treat infections [56,57]. The mechanisms by which resistance develops are complex and multifactorial, involving inappropriate prescription practices, the misuse of antibiotics in humans, animal health and agriculture, and micro-organisms [58,59]. The emergence of resistant strains is facilitated by antimicrobials in the environment, often at sublethal concentrations, leading to selective pressure on microbial populations, thereby encouraging the survival and proliferation of resistant variants [60,61]. Although AMR development is often associated with human activity, it is a natural phenomenon. Micro-organisms have innate mechanisms to protect themselves, such as enzymatic degradation of antibiotics, changes in cell wall permeability, and efflux pumps that expel antibiotics from the cells [62]. Furthermore, *Acanthamoeba* can protect intracellular bacteria from environmental stressors, disinfectants, and antibiotics [7,63], resulting in survival rates considerably higher than in free-living bacteria [63].

This study demonstrated that co-incubation of *P. putida* KT2440 with *Acanthamoeba* can lead to the development of high levels of resistance to ciprofloxacin and that the AMR pattern of the co-incubated bacteria differed significantly from that of the naïve lab strain. This study represents the first example of the induction of antibiotic resistance in a bacterial species co-incubated with *Acanthamoeba*. The latter has been recognized as a potential alternative to mammalian models for studying bacterial–host interactions because of its ubiquity, ease of culture, and functional similarities to human macrophages [6,64]. The interactions between *Acanthamoeba* and various microbes, including bacteria, can serve as a model for understanding the mechanisms of pathogenicity and host-pathogen interactions that are relevant to human diseases [6,9]. Further research is needed to investigate the molecular mechanisms underlying the development of resistance in co-incubated *P. putida* and to explore potential strategies for mitigating the spread of antibiotic resistance in environmental and clinical settings.

## Supplementary material

10.1099/acmi.0.000999.v3 Uncited Fig. S1.

## Abbreviations

AMR: antimicrobial resistance
AST: antibiotic susceptibility testing
CLSI: Clinical and Laboratory Standards Institute
EUCAST: European Committee on Antimicrobial Susceptibility Testing
LB: Luria–Bertani
MPC: mutant prevention concentration
PG: peptone glucose
PMF: proton motive force
QRDR: quinolone resistance-determining regions
