# Supplementary material for: Preliminary insights into the potential role of Acanthamoeba–Pseudomonas interactions in the development of antibiotic resistance
Source: Access Microbiol. 2025 Jun 30;7(6):000999.v3. doi: 10.1099/acmi.0.000999.v3 (PMC12281815; doi:10.1099/acmi.0.000999.v3)
Supplement: Uncited Fig. S1. [file acmi-7-00999-s001.pdf]

Supplementary Figure 1

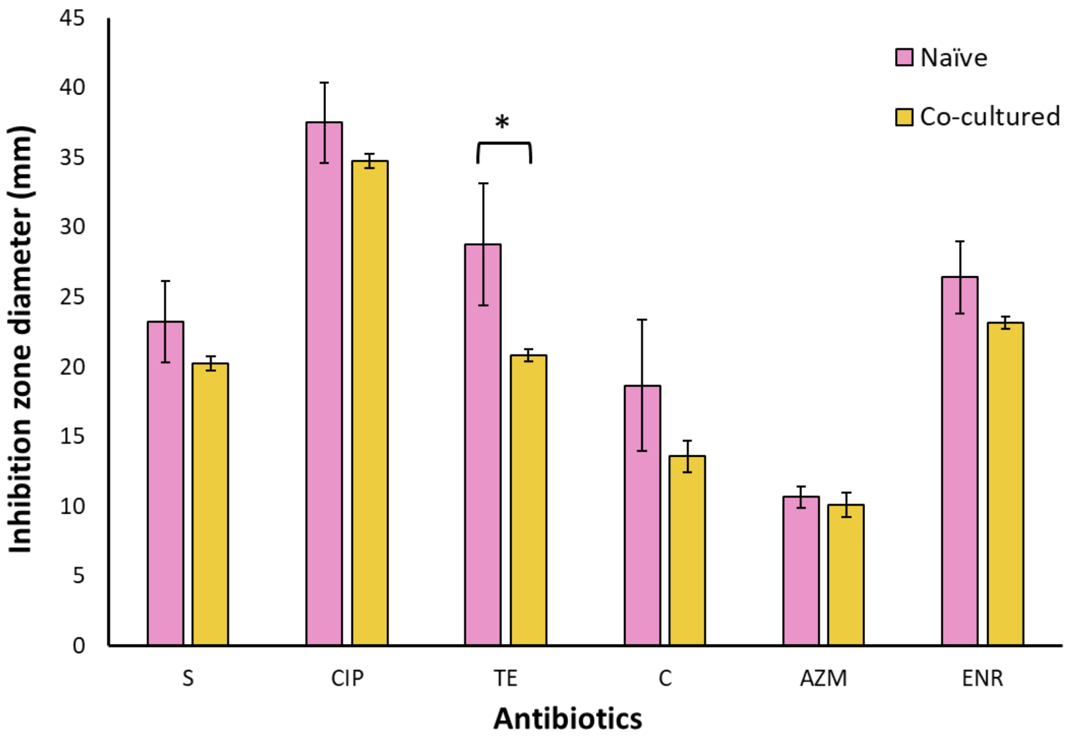

Figure S1. Resistance profiles of *P. putida* KT2440 not passaged within *Acanthamoeba* (naïve strain) and those passaged three times within *Acanthamoeba*. All cultures remained susceptible to all antibiotics tested, with only a slight decrease in susceptibility to tetracycline observed in the co-cultured cells (yellow bar) relative to naïve cells (pink bar). Statistical analysis was performed using Welch's t-test, with significance levels denoted as "*". Error bars represent SD. Data are presented as mean ± SD from four independent experiments, each performed in triplicate.
